# Functional results in airflow improvement using a “flip-flap” alar technique: our experience^☆^

**DOI:** 10.1016/j.bjorl.2017.01.006

**Published:** 2017-02-21

**Authors:** Arianna Di Stadio, Carlo Macro

**Affiliations:** a University La Sapienza, Rome, Italy; b Ospedale San Camillo-Forlanini, Dipartimento Chirurgia Maxillo-Facciale, Rome, Italy

**Keywords:** Alar cartilage, Nose point, Surgery, Rhinomanometry, Functional esthetic, Cartilagem alar, Ponta nasal, Cirurgia, Rinomanometria, Estética funcional

## Abstract

**Introduction:**

Pinched nasal point can be arising as congenital malformation or as results of unsuccessfully surgery. The nasal valve alteration due to this problem is not only an esthetic problem but also a functional one because can modify the nasal airflow. Several surgical techniques were proposed in literature, we proposed our.

**Objective:**

The purpose of the study is the evaluation of nose airway flow using our flip-flap technique for correction of pinched nasal tip.

**Methods:**

This is a retrospective study conducted on twelve patients. Tip cartilages were remodeled by means of autologous alar cartilage grafting. The patients underwent a rhinomanometry pre and post-surgery to evaluate the results, and they performed a self-survey to evaluate their degree of satisfaction in term of airflow sensation improvement.

**Results:**

Rhinomanometry showed improved nasal air flow (range from 25% to 75%) in all patients. No significant differences were showed between unilateral and bilateral alar malformation (*p* = 0.49). Patient's satisfaction reached the 87.5%.

**Conclusion:**

Our analysis on the combined results (rhinomanometry and surveys) showed that this technique leads to improvement of nasal flow in patients affected by pinched nasal tip in all cases.

## Resumo

**Introdução:**

A ponta nasal comprimida pode surgir como malformação congênita ou como o desfecho de uma cirurgia malsucedida. A alteração da válvula nasal devido a esse problema não é apenas um problema estético, mas também funcional, porque pode modificar o fluxo aéreo nasal. Várias técnicas cirúrgicas têm sido propostas na literatura; aqui, propomos a nossa.

**Objetivo:**

O objetivo do estudo é a avaliação do fluxo das vias aéreas nasais utilizando nossa técnica de *flip-flap* para correção da ponta nasal comprimida.

**Métodos:**

Este é um estudo retrospectivo realizado em doze pacientes. As cartilagens da ponta nasal foram remodeladas através de enxerto de cartilagem alar autóloga. Os pacientes foram submetidos à rinomanometria pré e pós-cirúrgica para avaliar os resultados, e realizaram uma auto-avaliação para avaliar seu grau de satisfação em termos de melhora da sensação do fluxo aéreo.

**Resultados e conclusão:**

Nossa análise dos resultados combinados (rinomanometria e auto-avaliação) mostrou que essa técnica melhora o fluxo nasal em pacientes afetados por ponta nasal comprimida em todos os casos. A satisfação do paciente atingiu os 87,5%.

## Introduction

A pinched nasal tip is often due to a deformity of the alar cartilage. It can be due to a congenital malformation or the results of previous rhinoplasty. This cosmetic deformity can be associated with nasal airflow obstruction as a consequence of internal nasal valve reduction. Hirschberg^1^ reported that the area of greatest airflow resistance is located in the first two centimeters into the nasal cavity, being responsible for 56% of nasal resistance in basal conditions and by 88% after using topical decongestant, it means that nasal valve is widely involved in this process. The cartilage deformity is able to create an alteration in the degree of the internal nasal valve. The nasal valve is formed by internal and external portions. The external valve is formed by the columella, the nasal floor, and the nasal rim (or the caudal border of the lower lateral cartilage). The internal nasal valve is located in the area of transition between the skin and respiratory epithelium and it is usually the narrowest part of the nose. The valve area is defined by the nasal septum, the caudal border of the upper lateral cartilage, the head of the inferior turbinate, and the pyriform aperture and tissues that surround it. This area is responsible for more than two-thirds of the resistance produced by the nose. In white Caucasians, the valve's angle is 10°–15°.

The nasal valve is the entry of nasal vestibule that has the shape of and acts as a tube joint, redirecting the air that comes from the front, below and the sides, thus creating a laminar flow.^2^ Nasal vestibule has thermo receptors that contribute to the feel of nasal patency.^3^ It means that the role of nasal valve and of the nose point it is more functional than esthetic, so in case of pinched nose the reduction of perceived and effective nasal airflow can create an important disease in affected patients.

Pinched nose tip is commonly treated surgically using cartilage grafting from the septum, ear, or rib, as well as internal sutures to treat the alar cartilage deformity. In this article, we propose a technique that uses autologous alar cartilage grafting to correct the pinched nasal tip. Floating flaps of alar cartilage have been used to remold and support the nasal tips, for example, by sliding the cephalic portion of the lower lateral cartilage under the caudal alar cartilage^4^ to obtain support. Our technique improved the tip contour and increased the internal nasal valve's diameter.

## Patients and methods

**Figure 1:**
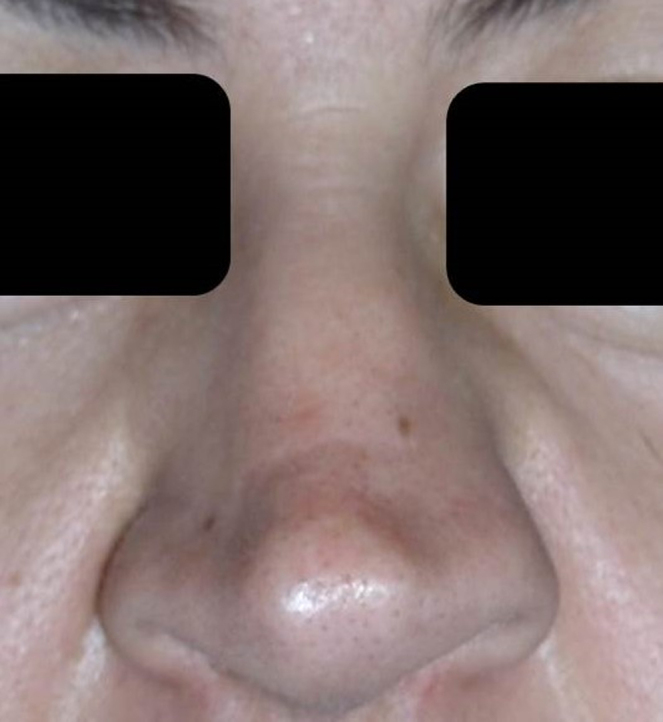
Pre-operatory patient photo.

A twelve case series is described in this report as a retrospective study (six males, six females, age range 25–50 years). This study was conducted in respect of Helsinki declaration and it was approved by Ethical Commission of the Institution as retrospective study without an IRB number, because this is the standard of Italian Hospital legislation in case of retrospective study. Treatments were performed from May 2009 to May 2010. Patients affected by nasal breathing insufficiency underwent nasal endoscopy to identify any associated septal deviations. Patients were divided in two groups based on the function of their alar cartilage deformity (Fig. 1). Group A included patients (7 cases) affected by unilateral alar cartilage asymmetry and Group B included patients (5 cases) with bilateral alar cartilage asymmetry.

We included in our study patients who underwent anterior active basal rhinomanometry (RHINOSPIR-PRO) before their nose surgery to study nasal airflow and without any septal deviation. We evaluated the patients with a 12 month follow up utilizing the same machine. We performed a simple survey to understand their level of nasal airflow sensation satisfaction before and after surgery. The satisfaction rating ranged from 1 to 3, with 3 as “fully satisfied”, 2 “quite satisfied”, and 1 “not satisfied”.

A two tailed *t*-test was performed to analyze the meaning of the data; *p* < 0.05 defined a significant value.

### Surgical technique description

**Figure 2:**
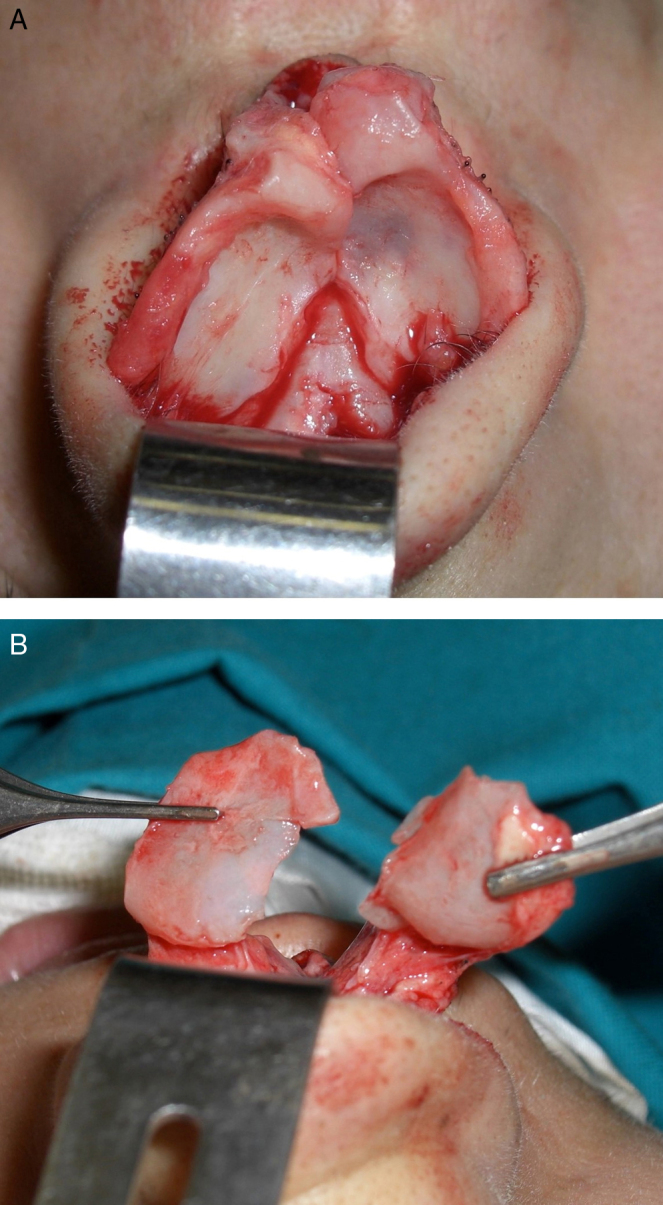
(A) Alar cartilage exposition after the open technique was performed. (B) Bilateral cut of the posterior cartilage portion.

It is possible to perform this technique in local anesthesia with soft patient's sedation as we did or in general anesthesia due to the patient’ necessities. A local infiltration injection with Mepivacaine 2% hydrochloride was performed at the level of the internal and external portions of the nasal tip so to obtain hydrodissection, vasoconstriction and local anesthesia at the same time. All patients underwent to an open rhinoplasty by a transcolumellar approach, taking care to the nasal mucosa preservation. An inverted V-shaped columellar incision was used; the incision was made within 2 marginal incisions in the nasal vestibule.^5^ The marginal incisions were made in very caudal position creating an accommodation site where cartilage will be re-inserted at the end of the surgery.^6^ The alar cartilages, carefully dissected, were completely exposed to visualize their abnormal anatomic insertions or/and deformities. The deformed alar cartilages were cut from the anterior (1 mm under the dome) to their posterior insertion (Fig. 2A and B). The incision totally separated the alar cartilages from their insertion points and enabled their complete removal from the original position. Removed cartilages were remodeled and shaped to achieve symmetry and correct normal anatomy.

**Figure 3:**
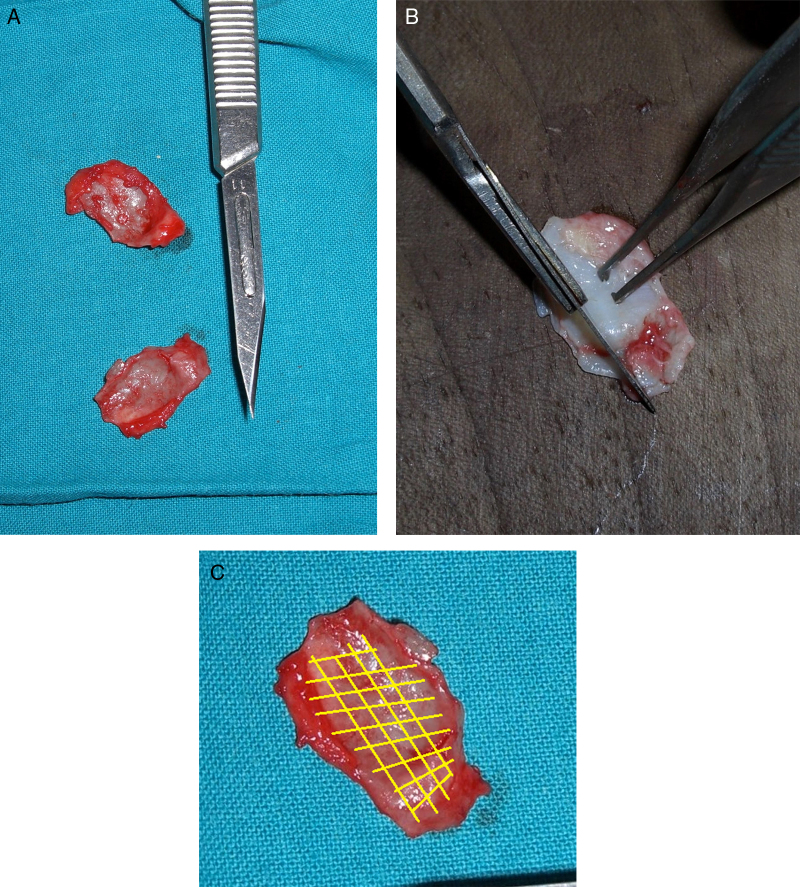
(A) Images of cartilages detached from the original point. (B) Remodeling of the cartilage to achieve the correct anatomy. (C) Fragilizing lines scheme.

**Figure 4:**
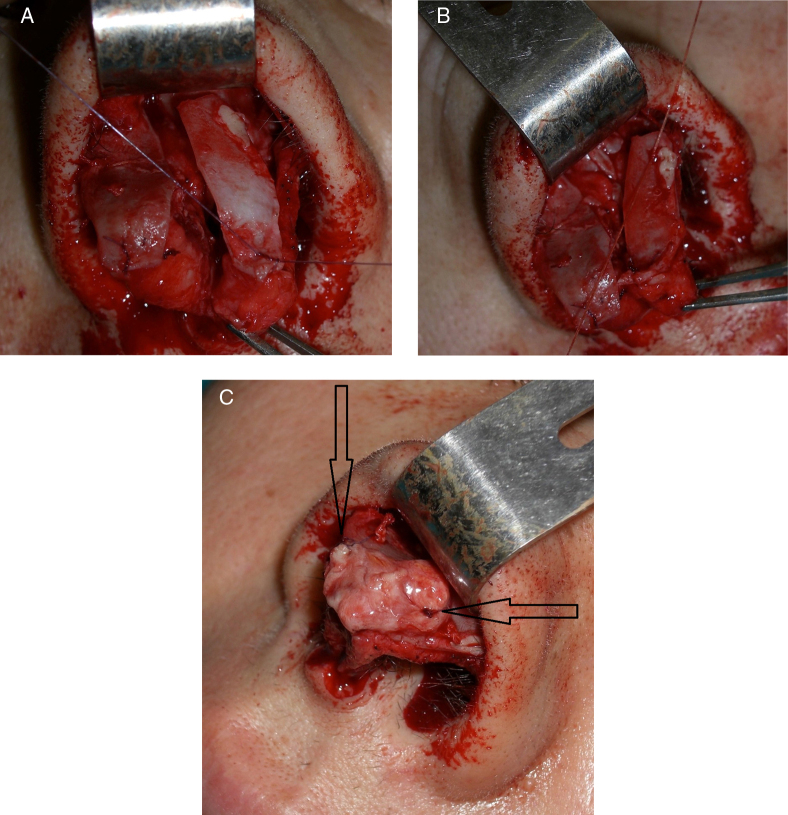
(A) Suture of the anterior portion of the alar cartilage. (B) Suture of the posterior portion of the alar cartilage. (C) Apposition of the columella graft to define the point, the arrows show in detail the sutures positions.

**Figure 5:**
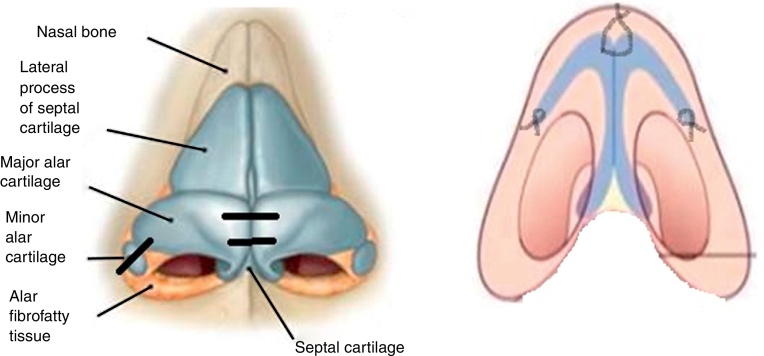
Details of suturing.

As first, the excess cartilage was cut in the cephalic portion of the alar cartilage and same was made for the inferior portion to reach a linear edge (Fig. 3A and B). Cartilage weakening incisions were made on the convex part of the deformed alar cartilages (in 3 cases, cartilage thinning was also necessary). The weakening incisions have to be numerous in vertical and horizontal sense on the cartilage to destroy the “cartilage memory”, fragilizing it without too much deepness avoiding irreversible damages of the structure (Fig. 3C). Turning the cartilages 180° by upside down inversion (flip-flap technique) then the cartilages were reinserted and stabilized by two central sutures with 6.0 nylon fixed on the dome (to address the anterior portion of the cartilage and reach out a tip definition). These sutures are placed following a small diagonal line, the needle is inserted before on the dome cartilage at 1.5 mm from is resected border, then placed on the remodeled alar cartage at 2.5 mm from its edge to obtain an slight overlapping of the cartilages, necessary to better stabilize the sutures as well as to better define the tip. The posterior portion of both cartilages was fixed to the dense soft connective maxillary tissue, restoring its original anatomic position, by two sutures 6.0 nylon (Fig. 4A and B); these sutures are placed deeper in the connective tissue avoiding the come out from the skin and because in this point the resistance is low without risk of needle damage. No any others sutures will be necessaries because during the healing repair the mucosa will naturally attached to the repositioned cartilage. The hyper-point tip projection and its definition were reached placing a single suture between the two nasal domes (3 cases) (Fig. 5). Where necessary (3 case in our study, see results section), a columellar strut graft was added over the tip cartilages to achieve greater tip support and shape regularization (Fig. 4C). The strut graft, where necessary, was modeled in a small triangle shape and fixed on the tip with two single suture one for each side of the graft (arrow in Fig. 4C). Marginal incisions were sutured by 5.0 Vycril rapid sutures; skin suture was performed using 5 points of Nylon 5/0. A Steril-Strip™ was applied in the end of surgery.

## Results

Tip surgery with a columellar strut graft was needed in 3 (25%) of the twelve cases only.

The survey results showed complete satisfaction in 50% of the cases (8 patients), 37.5% of the cases (3 patients) responded as quite satisfied and only one patient (12.5%) expressed dissatisfaction.

**Figure 6:**
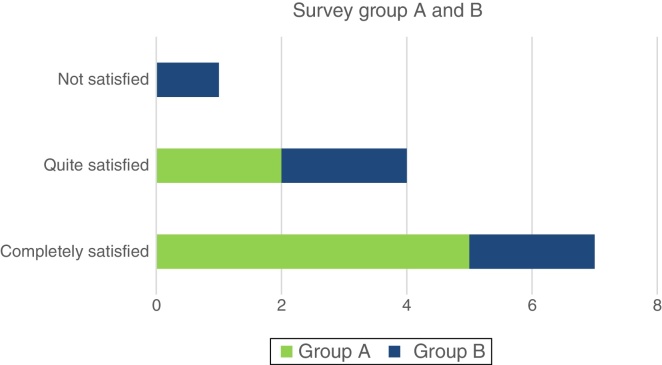
Results of survey.

The distribution of answers within the two different groups were: 5 patients in Group A and 2 patients in Group B responded as “completely satisfied”; 2 patients in Group A and 2 in Group B responded as “quite satisfied”; and 1 patient in Group B responded as “not satisfied” (Fig. 6).

**Figure 7:**
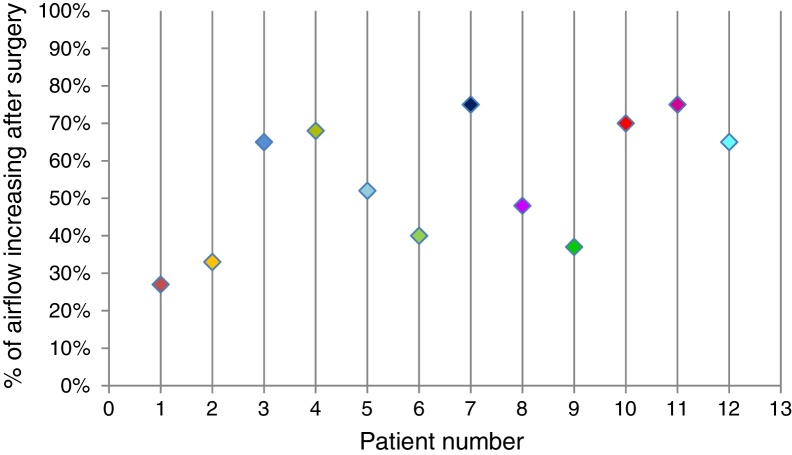
Diagram of patient increasing nasal air flow after surgery.

Rhinomanometry showed, in opposition to patient's answers, improved nasal air flow (range from 25% to 75%) in all patients (100% of cases) treated for alar cartilage deformity correction (Table 1 and Fig. 7).

**Table 1:** Increase of nasal air flow after surgery for each patient.

Rhinomanometry results	
Patient	Improvement
1	27%
2	33%
3	65%
4	68%
5	52%
6	40%
7	75%
8	48%
9	37%
10	70%
11	75%
12	65%

We compared the results of the rhinomometry variation pre and post-surgery between the unilateral group (A) and the bilateral (B) and no significant differences were showed between the two groups (*p* = 0.49).

## Discussion

Several techniques to treat nasal tip malformation have been described in the literature.^7^, ^8^ Our surgical procedure is a simple method that is able to treat alar cartilage malformations.

We propose a complete alar cartilage section and removal, in opposition to other proposed techniques,^9^ even if this is contrary to more common suggested, because we believe (following our results) that the technique is less aggressive than appears. Removal of the cartilages carefully dissected is not aggressive in expert hands, it is widely showed that an aggressive surgery modality even if made in closed technique^10^ is able to determine worse functional and esthetic outcomes that open/reconstruction technique.^11^ Sometimes an hard manipulation trying to reach the result using a conservative technique give worse results that a soft manipulation using technique that can appear more aggressive.^12^

Autologous cartilage is used in our in respect of the first fundamental principle of nasal reconstruction. Use of an autologous graft is important to avoid rejection and helps to obtain a natural result, especially on skin nose tip that is very thin. Our technique does not require the use of cartilaginous graft reinforcement to improve the internal nasal valve degree, unlike other techniques that have been proposed.^13^, ^14^

This option offers two advantages: there is no need for donor site cartilage and there is reduced risk of the collapse of the nasal valve with graft displacement.

The role of the internal nasal valve's diameter is well-known for its ability to influence nasal air flow^15^, ^16^ and the patient's perception of nose air flow too. For this reason, many authors proposed different options to solve the problem^17^ as insertion of graft to increase the nasal valve for example.

Physiologic air flow in the nose^18^ mostly goes through the lower portion of the nasal cavity, and so a small problem in this zone can appear as a large issue in terms of airflow perception. One example of this is how inferior-anterior septum deviation^19^ is able to cause serious distress in patients.

The patient's perception of nasal airflow can be tested using the Killian technique. Patients can understand the difference between small and large nasal valves, corresponding to a volume increase.

We reached a patient satisfaction rate of 87.5% in terms of functional results using our technique.

Limitation of the study: due to the small number of patients the results can be considered only preliminary. This technique is adapted to a senior surgeon able to treat tissue with softness.

## Conclusions

We re-proposed a surgical technique that is well established in literature, because we think that some details can do the difference in term of functional results and patient's satisfaction.

The cartilage fragilization that we made using a grill pattern, undoubtedly request time and care but give the better results in long term, avoiding the restore of the old cartilage shape.

The tip projection that we obtained placing the suture as described above – diagonal line – and the choice to improve, where necessary, the surgery results with a columellar strut graft are a relevant factor in good reached rhinomanometry results.

Our technique can be useful also in lateral cross deformity in patients affected by outcomes of cleft palate; in this case the association between the flip-flap and the columellar strut graft can offer good results.

Despite the limited number of treated patients, we believe that this simple-to-perform technique is a very good treatment option to correct a pinched nasal tip determined by monolateral or bilateral cartilage malformation, and it is important to reiterate that even if other authors presented it in the past, small variation can be done to improve more it. At least our functional results showed again how it is important the nasal valve in the breath physiology.

## Conflicts of interest

The authors declare no conflicts of interest.
